# Aerosol gas exchange system (AGES) for nanoparticle sampling at elevated temperatures: Modeling and experimental characterization

**DOI:** 10.1038/s41598-019-53113-5

**Published:** 2019-11-20

**Authors:** Markus Bainschab, Sampsa Martikainen, Jorma Keskinen, Alexander Bergmann, Panu Karjalainen

**Affiliations:** 1 Graz University of Technology, Insititute of Electronic Sensor Systems, Graz, 8010 Austria; 2 Tampere University, Aerosol Physics Laboratory, Tampere, 33720 Finland

**Keywords:** Characterization and analytical techniques, Characterization and analytical techniques, Atmospheric chemistry

## Abstract

An aerosol gas exchange system (AGES) for nanoparticle sampling at elevated temperatures was developed, modeled, and further characterized with laboratory tests with respect to gas exchange efficiency and particle losses. The model describing the gas exchange was first verified with oxygen and later studied with several inert gases having molecular masses between 18 and 135 u. The exchange rate of the lightest compounds exceeds 90% efficiency at the flow rates used. In order to reach similarly high removal efficiencies for larger molecules, the residence time in the AGES has to be increased. The removal of sticky gases was studied with gaseous sulfuric acid. Results agreed with the model where the boundary condition is zero concentration on the wall. The AGES exhibits very limited particle losses (<5%) for mono-disperse 6 nm particles. Furthermore, diffusional losses for particles down to 1.2 nm were measured utilizing polydisperse aerosol. The experimental findings are in good agreement with the model derived. As both, gas exchange rate and particle losses, rely on the physical effect of diffusion, an optimization for enhanced gas exchange efficiency will come at the cost of increased diffusional particle losses. The presented model can be used as a tool to redesign and optimize the AGES for a desired application. With an application targeted design, particle dilution can be avoided, which can lead to improved results in many fields of aerosol measurement.

## Introduction

The quality of measurement results in aerosol studies is often not limited by the applied measurement instrument but by uncertainties introduced in the course of the sampling procedure. Sampling is especially difficult in the presence of the smallest nanoparticles and when the aerosol contains a large quantity of semi-volatile particulate matter that can be found in either gaseous or condensed states depending on sampling conditions, e.g. residence time and temperature in the sampling system. These extremely challenging aerosol measurement fields include for example engine emission studies^1–4^ and atmospheric studies^5–7^. The sampling phase must (1) reduce particle concentrations to levels suitable for the measurement instruments, and (2) prevent condensation of gases on the surfaces of the sampling system itself or inside the measurement instruments. Often particles of a certain degree of volatility are of special interest. In engine emission studies, the fraction of particles that do not evaporate below 350 °C are most commonly the focus^8^. This focus sets additional requirements to the sampling procedure because the particle fraction of interest has to be isolated. However, existing methods for the isolation of non-volatile particles exhibit major drawbacks. This work presents an alternative method that can overcome many drawbacks of the existing solutions and is applicable to different fields of aerosol measurement.

Until recently, the type approval of vehicles required the determination of particulate mass emissions over a test cycle. The particle emissions were measured from the filter mass increase when diluted vehicle exhaust was collected on a paper filter. The introduction of diesel particulate filters (DPF) reduced emitted particle mass concentrations to levels where they can no longer be reliably measured gravimetrically. This made the transition to alternative particle measurement methods and metrics inevitable. Based on the studies of the Particle Measurement Programme (PMP) group (a programme managed by the UN-ECE), a methodology was introduced in the European legislation to count non-volatile particles – those that are not vaporized at 350 °C– of diameter >23 nm from diluted exhaust over a specific test cycle. Recently, the PMP methodology was extended to assess real driving emissions (RDE) of particle number (PN) by Portable Emission Measurement Systems (PEMS), mounted on vehicles in real-world conditions. In this case, measurements must be conducted for practical reasons from the raw exhaust. While requirements exist for legislative measurements regarding sampling and transport of the aerosol sample, such requirements do not necessarily allow appropriate quantification of particle number emissions from raw exhaust measurements^8^. This is particularly prevalent for particle sizes smaller than 23 nm and especially below 10 nm. To reach the so called non-volatile particle fraction, heated sampling systems like thermodenuders (TDs)^9,10^ or catalytic strippers (CSs)^11,12^ have frequently been applied after sample dilution. TDs collect gases on the surface of activated charcoal, hence they require regular service. In the CS, gaseous organic compounds are oxidized and sulphuric acid is stored and thus these also require service and regeneration. Both TD and CS systems efficiently remove semi-volatile material, but due to their large surface areas, they often induce high losses of sub-10 nm particles. The current PMP protocol relies only on heated sampling and dilution to measure particles larger than 23 nm^8^, and in the near future down to 10 nm. Maintaining comparable levels of accuracy in future regulatory particle number measurements would most likely require sampling systems that actively remove volatile compounds while exhibiting limited diffusional particle losses. This means that alternative exhaust conditioning approaches, apart from the existing CS, TD and the PMP method, are needed.

As an example, a concept of a counter flow denuder (CoFD) was recently published, where the working principle consists of an exchange of the carrier gas of an aerosol sample through diffusion across a cylindrical porous glass tube to a purge gas flow^13^. In the CoFD, the removal efficiency of gases increased with a lower sample flow rate and a higher purge-to-sample gas flow rate ratio. The pore size of the micro-porous glass did not affect the gas removal efficiency and particle penetration efficiency. High particle penetration (94% penetration for 20 nm particles) was observed experimentally. The values agreed with the theoretical estimation of diffusion losses.

In this work, a complete sampling system was constructed using the principles of CoFD. In addition, adjacent heaters, flow control, and a data collection system were implemented. The applicability of this aerosol gas exchange system (AGES) tolerating about 200 °C was tested with a synthetic laboratory aerosol. In the tests, both gas exchange efficiencies and particle losses were studied. Gas exchange efficiencies were studied using several gases of varying molecular masses. The particle losses were characterized down to 1.2 nm with Ag nanoparticles. A model describing the performance was derived, calibrated and experimentally validated.

The AGES system is a novel approach to the world of engine exhaust aerosol measurement, allowing robust quantification of particle properties down to the nanometre regime for research as well as legislative purposes. Apart from the application in exhaust particle measurements, the AGES can also allow for improved results in other fields of aerosol measurement. One example is the assessment of chemical properties of aerosol nanoparticles, where a separation of particles and carrier gas or comprehensive knowledge about the composition of the carrier gas is a requisite. Recently a method was presented that relies on dilution with argon to enable the application of an inductively coupled plasma mass spectrometer (ICP-MS) for the size-resolved elemental analysis of nanoparticles^14,15^. Carrier gas exchange instead of dilution would yield higher particle concentrations at the measurement instrument and hence a reduced limit of detection.

The operation parameters of the AGES can be tuned to meet the requirements of specific applications. This enables the AGES to act as a standalone sampler for aerosol instruments because all the gaseous impurities from the original exhaust can be removed in a single system. This opens new opportunities to sampling from exhaust flows containing very low levels of particulate matter at very low particle sizes like those from gas engines, jet engines or downstream of a particulate filter (PF).

## Instrument Description

**Figure 1:**
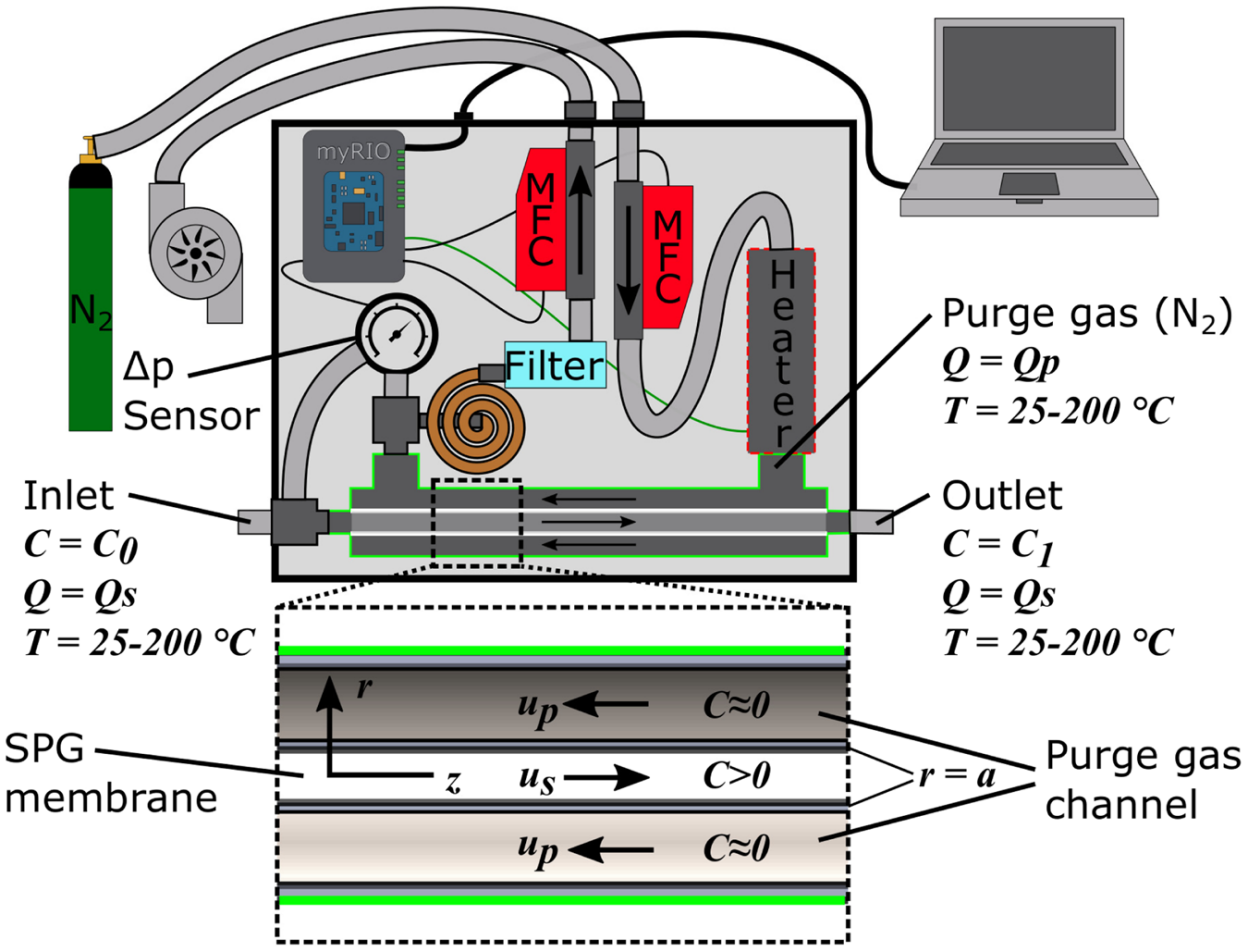
Drawing of the aerosol gas exchange system. The purge gas flow (N2) is controlled with a Vögtlin red-y mass flow controller. The pressure difference between the sample gas channel and the purge gas channel was measured with a Honeywell TSCSNBN005PDUCV. The controllable valve of another Vögtlin red-y MFC is programmed to keep the pressure difference as low as possible (typically <0.5 mbar). The part highlighted with a green frame represents the counter flow denuder. *u*_*p*_ and *u*_*s*_ are the flow velocity in the purge gas channel and the sample channel respectively. *C*_0_ and *C*_1_ are the concentrations of the substance to be removed at the inlet and the outlet of the system.

The AGES can be applied for removing gaseous compounds of an aerosol at temperatures up to 200 °C. The system’s center piece is a counter flow denuder (CoFD)^13^. This device has previously been shown to enable a non-specific removal of gases from an aerosol while exhibiting fractional particle losses below 6% for polystyrene latex (PSL) particles larger than 20 nm. Gas molecules in the sample flow are exchanged by diffusive transport through a Shirasu Porous Glass (SPG, SPG Techno Co. Ltd., Japan)^16^ membrane with the shape of a hollow cylinder. The membrane has an outer diameter of 5 mm, a wall thickness of 0.4 mm, a mean pore size of 100 nm and an active length of 200 mm. The anti-parallel flow directions of the the purge gas flow and the sample flow provide a high concentration gradient for the whole length of the denuder. Therefore, gas removal efficiencies close to 100% can be reached. To make the counter flow denuder applicable for challenging environments it is necessary to control the pressure difference between the sample flow line and the purge gas line and to control the temperature of the purge gas flow. A pressure difference between the sampling line and the purge gas line induces a flow through the porous glass membrane. Therefore the pressure difference has to be kept as low as possible to ensure that diffusion dominates the convective mass transport through the porous glass membrane. A pressure control unit was implemented in the AGES to control the pressure difference between purge gas line and sample line. A sensor (Honeywell TSCSNBN005PDUCV) measures the pressure difference between purge gas line and the sample line. A LabVIEW virtual instrument running on a NI myRIO takes the pressure difference as an input parameter for a PID controller that controls the valve of a mass flow controller to minimize the pressure difference. Figure 1 shows a drawing of the aerosol gas exchange system.

### Mathematical model

In this section an analytical model is derived to find an approximate form of the removal efficiency as a function of operation parameters and species. The results of a laboratory experiment are used to find the membrane material and flow profile specific parameter *R*_m_^*^ to adjust the derived general model for the specific denuder.

A mathematical expression for the removal efficiency of the counter flow denuder can be found in the literature^13,17^. However, the equation shown there is not generally applicable for the description of the presented type of denuder. Mathematically speaking, the reason why this equation cannot be applied for the description of the CoFD is that the boundary conditions do not match the conditions the solutions provided by several groups are based on^18–20^. The provided equations are based on the Dirichlet boundary condition that the concentration *C* of the substance to be removed is zero at the wall of the denuder (*r* = *a*).

1 $$C(r=a)=0$$

This condition only holds for gaseous species that are adsorbed at the porous glass membrane, and for particles that stick to the wall upon getting in contact with it. The transport of gaseous substances that are not absorbed by the glass membrane is described correctly with a Robin boundary condition. The diffusive mass flux *J* through the porous membrane of these substances is proportional to the difference between the concentrations *C*_*s*_ and *C*_*p*_. *C*_*s*_ is the concentration at the inner wall of the membrane, which limits the sample channel. *C*_*p*_ is the concentration at the outer wall of the membrane, which is the boundary of the purge gas channel.

2 $$J(r=a)=D{\frac{\partial C}{\partial r}|}_{r=a}=\alpha ({C}_{s}-{C}_{p})$$

*D* is the diffusion coefficient of the substance to be removed. The proportionality factor *α* depends on the diffusion resistance *R*_*m*_ and the convection coefficient *h*_*m*_, which describes the effectiveness of the convective transport in the purge gas channel. The diffusion resistance *R*_*m*_ is the thickness of the porous membrane *d*_*m*_ divided by the diffusion coefficient of the substance to be removed in the porous membrane *D*_*m*_. The convective transport in the the purge gas channel dominates the diffusive transport through the membrane (*Q*_*p*_ = 8 sl min^−1^, Péclet number *Pe* > 5000) and the the bulk diffusion coefficient *D* is linearly proportional to the diffusion coefficient in the porous membrane because for the mean pore size of 100 nm constriction effects can be neglected^21,22^. Consequently, *α* can be assumed to be directly proportional to *D* in good approximation for the system described.

3 $$\alpha =\frac{1}{{R}_{m}+\frac{1}{{h}_{m}}}=\frac{1}{\frac{{d}_{m}}{{D}_{m}}+\frac{1}{{h}_{m}}}\propto D$$

The mass transport of substances that are not absorbed by the porous glass membrane are mathematically described by the convection-diffusion equation in cylinder coordinates (Eq. (4)) and the boundary condition in Eq. (2). Convection in *r*-direction is neglected because the sample flow velocities *r*-component is zero. Diffusion in *z*-direction is neglected because convection dominates the transport in *z*-direction (*Pe* > 1000) for typical values of the sample flow *Q*_*s*_ in the AGES.

4 $$\frac{1}{r}\frac{\partial }{\partial r}(r\frac{\partial C}{\partial r})+\frac{{u}_{z}}{D}\frac{\partial C}{\partial z}=0$$

where *u*_*z*_ is the flow velocity in *z*-direction.

The mathematical problem as described above cannot be solved analytically. In order to provide an approximate mathematical expression for the removal efficiency *RE*, the analytical solution of a very similar but slightly simpler problem is adapted. Crank^23^ provides a time dependent solution for a one-dimensional diffusion problem of the form:

5 $$\frac{1}{r}\frac{\partial }{\partial r}(r\frac{\partial C}{\partial r})=0$$

and the boundary condition in Eq. (2). This equation describes the counter flow denuder for 0 sample flow rate. The removal efficiency *RE* of this system after time *t* can be written as^23^:

6 $$RE=1-\frac{{C}_{1}}{{C}_{0}}=1-\mathop{\sum }\limits_{n=1}^{\infty }\,\frac{4{L}^{2}\exp (\,-\,{\beta }_{n}^{2}Dt/{a}^{2})}{{\beta }_{n}^{2}({\beta }_{n}^{2}+{L}^{2})}$$

where *β*_*n*_ are the solutions of:

7 $$\beta {J}_{1}(\beta )-L{J}_{0}(\beta )=0\,{\rm{with}}\,L=\frac{a\alpha }{D}$$

and *J*_*i*_ are the Bessel functions of the first kind of order *i*.

In order to adapt this solution to the counter flow denuder we first assume plug flow and later account for this simplification by modifying the parameter *R*_*m*_ that describes the diffusion resistance of the porous glass tube. Plug flow means that the flow velocity is equal at all points of the sample channel. As convection is the dominant transport mechanism in *z*-direction, diffusion in this direction can be neglected. Consequently, the residence time *t* in the counter flow denuder can be expressed in terms of the volumetric sample flow rate *Q*_*s*,*vol*_, the length of the SPG membrane *l* and the inner radius of the SPG membrane *a*:

8 $$t=\frac{l}{v}=\frac{l{a}^{2}\pi }{{Q}_{s,vol}}$$

According to Eq. (8), Eq. (6) can be reformulated as a function of the dimensionless parameter *μ*.

9 $$RE=1-\mathop{\sum }\limits_{n=1}^{\infty }\,\frac{\exp (\,-\,{b}_{n}\mu )}{{c}_{n}}\,{\rm{with}}\,{b}_{n}={\beta }_{n}^{2}\pi ;{c}_{n}=\frac{4{L}^{2}}{{\beta }_{n}^{2}({\beta }_{n}^{2}+{L}^{2})}\,{\rm{and}}\,\mu =\frac{Dl}{{Q}_{s,vol}}$$

To account for the simplified flow profile in the analytical model accompanied by an increased removal efficiency, the *R*_*m*_ is replaced by the modified parameter *R*_m_^*^. This parameter is an effective diffusion resistance, which is the material parameter *R*_*m*_ plus an additional resistance *R*_*flow*_ to account for the laminar flow profile.

10 $${R}_{m}\to {R}_{m}^{\ast }={R}_{m}+{R}_{flow}$$

The transition from *R*_*m*_ to *R*_m_^*^ is motivated by the similarity of the velocity and concentration profiles in the sample channel. Both profiles have their maximum at the center line of the sample flow channel and decay towards the wall. Because of this overlap the mean residence time of molecules in the denuder is reduced compared to a device with plug flow conditions. Consequently, the probability of molecules reaching the SPG wall within the length of the denuder is decreased. This decrease in probability can be described as a diffusion resistance which is added to the material specific diffusion resistance *R*_*m*_. It has been shown and applied successfully that kinetic resistances can be added to describe kinetic processes^24,25^. *R*_m_^*^ is determined experimentally and *R*_*m*_ and *R*_*flow*_ are derived from CFD simulations.

#### Model calibration

**Figure 2:**
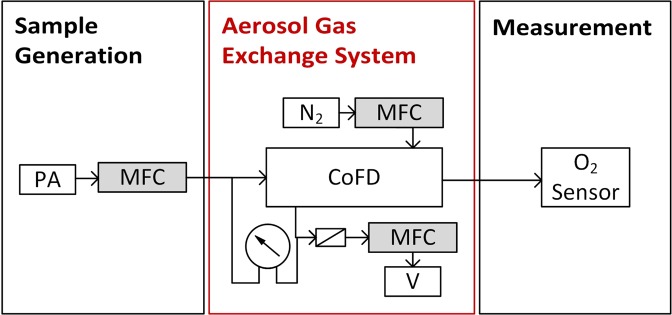
Schematic drawing of the experimental setup for the O_2_ removal measurements to find the diffusion resistance *R*_*m*_ of the SPG membrane. The sample flow (air) is controlled with a Vögtlin red-y mass flow controller. The O_2_ concentration at the outlet of the AGES is measured with an AlphaSense O2 A2 sensor.

In order to proof the validity of the mathematical expression derived, an experiment was performed. The results of this experiment are used to find the parameter *R*_m_^*^ that describes the diffusion resistance of the SPG membrane and accounts for the laminar flow profile in the sample flow channel. The removal efficiencies *RE* of O_2_ ($D=0.2\,{{\rm{cm}}}^{2}\,{{\rm{s}}}^{-1}$) were measured for different sample mass flow rates (standard conditions: *T* = 20 °C, *p* = 1013 hPa) at room temperature. The acquired data is used to fit the parameter *R*_m_^*^. The dependency of the removal efficiency of *R*_m_^*^ is described in Eqs (2, 3, 7, 9, and 10). Figure 2 shows a drawing of the experimental setup. Air was used as sample gas and nitrogen was used as a purge gas.

**Figure 3:**
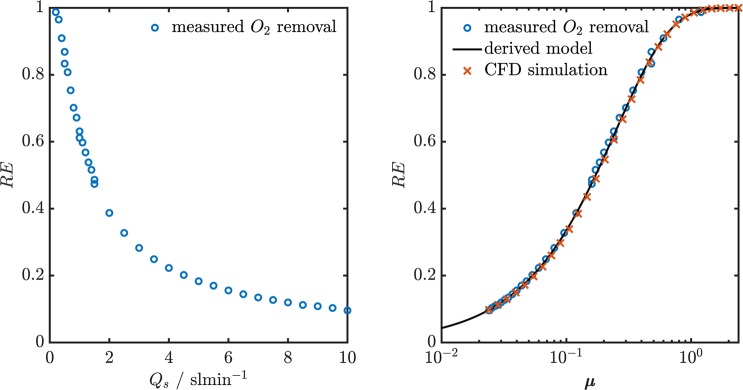
Left: Removal efficiency *RE* of O_2_ as a function of the sample flow rate *Q*_*s*_. Right: Measured O_2_ removal efficiency, derived model and CFD simulation results as functions of the dimensionless parameter $\mu =\frac{Dl}{{Q}_{s,vol}}$. The derived model uses *R*_m_^*^ = 137.9 sm^−1^ as effective diffusion resistance. The CFD simulations were performed taking *R*_*m*_ = 95.2 sm^−1^ as diffusion resistance of the porous glass tube.

The nitrogen purge gas flow was set to 8 sl min^−1^. The oxygen concentration in the pressurized air and the outlet of the AGES were measured with an electrochemical AlphaSense O2 A2 sensor. This measurement was repeated for flows between 0.2 sl min^−1^ and 10 sl min^−1^. Figure 3 shows the O_2_ removal efficiency as a function of the sample flow rate *Q*_*s*_ on the left and the removal efficiency as a function of *μ* on the right.

The sample flow measurement points are converted to values of *μ* before fitting the function in Eq. 6 with the parameter *R*_m_^*^. The right plot in Fig. 3 shows the result of this fit. It can be seen that the derived model fitted with only one parameter agrees with the measured data very well over a large range of removal efficiencies (from under 10% to over 90%. This result shows that the simplification of the problem and the inclusion of the effect of the laminar flow profile in the effective diffusion resistance *R*_m_^*^ is an appropriate way to find a mathematical expression for the removal efficiency as a function of *μ*, which can be used to calculate the removal efficiency of different gases under different operation parameters. Only considering the first term of the infinite sum in Eq. 9 yields an approximation for the removal efficiency that deviates less than 0.01 from the infinite sum for all values of *μ* for the presented geometry and materials:

11 $$RE=1-0.990\ast \exp (\,-\,3.982\,\mu )$$

#### Computational fluid dynamics simulation

A 2D axismmetric model of the 20 cm long counter flow denuder was set up in COMSOL to simulate the oxygen removal laboratory experiment and to determine the diffusion resistance *R*_*m*_ of the porous membrane. The simulated geometry consisted of three rectangular domains each having a length of 20 cm. The innermost domain (*r* < 2.1 mm) represents the sample flow channel. The middle domain (2.1 < *r* < 2.5 mm) represents the porous glass membrane. In this domain the diffusion coefficient of the transported species is reduced to account for diffusion resistance *R*_*m*_ of the material. The modified diffusion resistance *R*_m_^*^ determined experimentally is used as an initial value for *R*_*m*_. The outermost channel (2.5 < *r* < 5 mm) represents the purge gas channel. The Reynolds number in the sample channel at a flow rate of *Q*_*s*_ = 1 sl min^−1^ is *Re* = 282, so the flow can assumed to be laminar at typical operation conditions of the counter flow denuder. Consequently, sample flow and the purge gas flow are simulated with the laminar flow interface. A no slip condition (**u** = 0) is applied at the interfaces of the three domains and the outer boundary of the purge gas domain. The purge gas flow rate is set to 8 sl min^−1^. The sample flow rate *Q*_*s*_ is varied between 0.001 sl min^−1^ and 10 sl min^−1^ with 15 steps per decade. The transport of the oxygen is simulated using the transport of dilute species interface, which includes diffusion and convection as transport mechanisms. The oxygen concentration *c*_0_ at the inlet of the sample channel is set to a normalized value of 1 mol m^−3^. A stationary study is performed for all values of *Q*_*s*_. The removal efficiency at each point of *Q*_*s*_ is evaluated. The described simulation is repeated for different values of *R*_*m*_ to find the value that leads to the most accurate reproduction of the experimentally found behavior. The value that fulfills this condition is found to be *R*_*m*_ = 95.2 sm^−1^. The results of the simulations with this value of *R*_*m*_ are shown in Fig. 3. The difference of the experimentally determined effective diffusion resistance *R*_m_^*^ = 137.9 sm^−1^ and the membrane diffusion resistance *R*_*m*_ = 95.2 sm^−1^ is assigned to the diffusion resistance induced by the laminar flow profile *R*_*flow*_ = 42.7 sm^−1^.

## Results

The data gathered in the experiments described in the methods section was evaluated to determine the AGES’ performance in terms of exchange efficiency for different gaseous species and particle losses. The gas exchange efficiency is represented as a function of the molecular mass of the species’ molecular mass and the dimensionless parameter *μ*. The particle losses are evaluated in two particle size regimes covered by the two different experimental approaches. Losses below 3 nm are determined from the data measured with the PSM whereas the losses between 6 nm and 23 nm are covered with the DMA-CPC approach.

### Gas exchange

**Figure 4:**
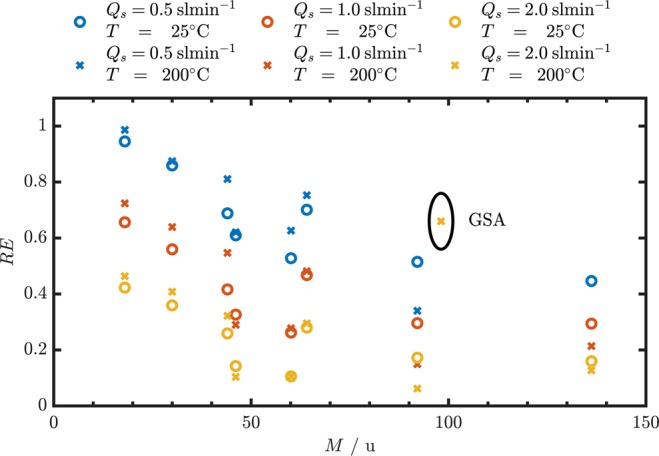
Gas removal efficiencies for the components listed in Table 1 as a function of the molecular mass at different temperatures and sample flow rates.

Figure 4 shows the removal efficiency of the tested substances listed in Table 1 as a function of the molecular weight. The removal efficiency was measured at three different sample flow rates at room temperature and at 200 °C with the exceptions of gaseous sulfuric acid (GSA, M = 98.1 u) where only measurements at elevated temperatures were performed.

**Table 1:** Table of substances tested listing the respective molecular weights *M*, the diffusion coefficients *D* at 25 °C and 200 °C as well as the measurement instrument used and typical concentrations upstream the AGES of the respective substance.

Substance	Chemical Formula	*M*/u	*D*/cm^2^ s^−1^ @25 °C	*D*/cm^2^ s^−1^ @200 °C	Typical Upstream AGES Concentration	Measurement Instrument
Water	H_2_O	18.0	0.30	0.66	20000 ppm	LI-COR
Nitric oxide	NO	30.0	0.20	0.40	3000 ppm	SIDOR
Oxygen	O_2_	32.0	0.20	0.40	21%	AlphaSense O2 A2
Carbon dioxide	CO_2_	44.0	0.15	0.31	100000 ppm	LI-COR
Sulfur dioxide	SO_2_	64.1	0.13	0.25	100 ppm	SIDOR
Ethanol	C_2_H_5_OH	46.1	0.12	0.23	3000 ppm	FID
Isopropanol	CH_3_CHOHCH_3_	60.1	0.10	0.20	3000 ppm	FID
Toluene	C_7_H_8_	92.1	0.09	0.17	4000 ppm	FID
Sulfuric acid	H_2_SO_4_	98.1	0.08^34^	0.18	7 × 10^8^ molecules/cm^3^	API-TOF
*α* pinene	C_10_H_16_	136.2	0.07^†^	0.13	4000 ppm	FID

^†^Indicates that the diffusion coefficient was calculated from Lenard-Jones potential parameters^35,36^. Values without superscripts are looked up and evaluated for 25 °C using the Chapman-Enskog theory^36,37^.

The highest removal efficiency observed was 99% measured for H_2_O at 200 °C and a sample flow of 0.5 sl min^−1^. The lowest efficiency value was 6% measured for toluene (*M* = 92.1 u) at 200 °C and a sample flow rate of 2.0 sl min^−1^. As expected, there is a trend that the removal efficiency rises with lower molecular masses. This is due to the fact that the molecular mass is indirectly proportional to the diffusion coefficient of the respective gaseous substance. The data points at *M* = 98.1 u, which represent sulfuric acid are an exception to this general trend. The reason is the high probability of sulfuric acid molecules to adsorb to surfaces.

Also the SO_2_ data points at *M* = 64.1 u protrude from neighboring data point measured under the same conditions. This behavior can be assigned to the relatively high diffusion coefficient of *D* = 0.13 cm^2^ s^−1^ given the molecular weight of *M* = 64.1 u of SO_2_. Ethanol for example has a substantially lower molecular weight (*M* = 46.1 u) but also a lower diffusion coefficient (*D* = 0.12 cm^2^ s^−1^) at room temperature.

Figure 4 clearly demonstrates that the removal efficiency rises when the sample flow rate is reduced. This statement is valid for all sets of measurements. For the example of H_2_O at room temperature a decrease of the sample flow rate from 2 sl min^−1^ to 0.5 sl min^−1^ increases the removal efficiency from 42% to 95%. Depending on the application, not the removal efficiency but the penetration can be the measure of interest. For this example an increase of the sample flow rate by a factor of 4 leads to an increase of the relative penetration by a factor of 11.6.

**Figure 5:**
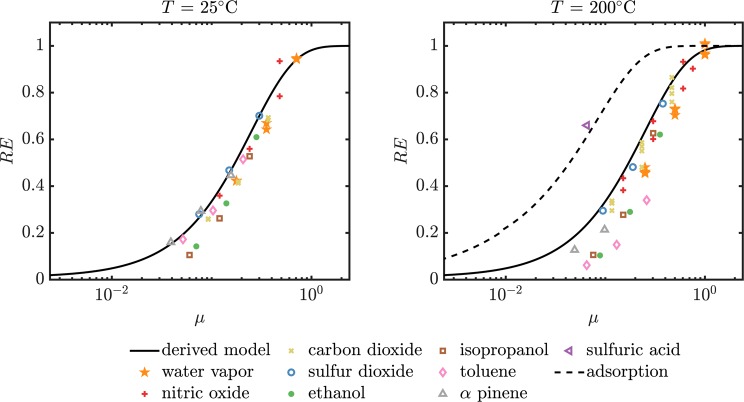
Removal efficiencies calculated from the measured upstream and downstream concentrations as a function of *μ* for different gaseous compounds at different flow rates. Left: Room temperature (25 °C experiment, Right: Experiment at elevated temperature (200 °C).

Another general trend that can be observed is the increased efficiency at elevated temperature. This behavior is reasonable because the increased diffusion coefficient at high temperatures (∝*T*^≈1.5^) overcompensates the decreased residence time due to a higher volumetric flow rate (∝*T*^−1^) at a constant mass flow rate. However, toluene (*M* = 92.1 u) and *α*-pinene (*M* = 136.2 u) do not follow this trend. For these two substances the observed removal efficiency at room temperature exceed the values measured at elevated temperature. Figure 5 shows the measured removal efficiencies at room temperature (left) and elevated temperature (right) as a function of the dimensionless parameter $\mu =\frac{Dl}{{Q}_{s,vol}}$. This representation facilitates the comparison of experimental data gathered under different conditions and the predictions of the theoretical model. The temperature dependence of the volumetric flow rate and the diffusion coefficients are accounted for in the calculation. For the sake of clearness the uncertainty in *μ* caused by the uncertainty of the sample flow measurement is not represented in the form of error bars. The relative uncertainties in *μ* amount to 10%, 5% and 2.5% for the data points measured for sample flows of 0.5 sl min^−1^, 1.0 sl min^−1^ and 2.0 sl min^−1^ respectively.

It is obvious at first glance at the two plots in Fig. 5 that the chosen experiment parameters are very suitable for the characterization of the aerosol gas exchange system, because the evaluated removal efficiencies are spread over a large range (6–99%). The removal efficiency shows a large dependence on *μ* in the experimentally covered range of *μ*. This allows for a comprehensive comparison with the efficiencies that are predicted by the theory. Hagino evaluated the removal efficiency under experimental conditions that lead to relatively high values of *μ*. This is the reason why removal efficiencies ≥89% for all investigated substances are reported^13^. Consequently, the fact that the theoretical model applied is not suitable was not conspicuous because the applied model also predicts removal efficiencies close to 100% for the respective values of *μ*. In the plot for the data collected at room temperature, all experimental data points are reasonably close to the line that represents the theoretical prediction (<10% deviation). However, the majority of the measured efficiencies are located below the theoretical efficiencies. The plot that shows the efficiencies at elevated temperature shows similar behavior but a higher deviation from the theoretical efficiency. A reason for this higher deviation can be that the sample line was not perfectly isothermal due to heat losses. The point representing GSA shows a significantly higher removal efficiency than other substances at comparable values of *μ*. The dashed black line in the plot shows the theoretical removal efficiency for substances that adhere to the wall of denuder^17^. This is the curve used by Hagino^13^ to predict the performance of the counter flow denuder for all substances. The experimental data shows that the assumption of gas molecules adhering to the wall is only valid for sulfuric acid amongst the test substances. For the case of wetted surfaces there are strongly varying values of the GSA mass accommodation or “sticking” coefficient reported in the literature^24,26,27^. However, due to the very large surface area of the porous glass tube we can assume here that this “sticking coefficient” is very close to unity for GSA in our device. This means that it can be assumed that a GSA molecule adheres to the surfaces similarly to condensation, when it gets in contact with the membrane. For the majority of compounds the boundary condition described in Eq. 2 has to be applied and consequently the calculation of the removal efficiency has to be performed using the formula in Eq. 6.

The plot in Fig. 5 showing the data of the experiment at 200 °C shows that the agreement of the measured data with the theoretical behavior is not of equal quality for all substances investigated. The CO_2_ removal efficiencies measured at different flow rates agree well with the behavior predicted by the model. For H_2_O the removal efficiency at a sample flow rate of *Q*_*s*_ = 0.5 sl min^−1^ (*μ* = 1.0) agree well with theory but the deviation increases for higher flow rates. The observation made in Fig. 4 that the removal efficiency of toluene and *α*-pinene at elevated temperatures are lower than expected is evident also in Fig. 5. Also the measured removal efficiencies of isopropanol and ethanol show a relatively high deviation to the values predicted by the model. In general it can be said the the model derived overestimates the removal efficiencies of hydrocarbons at high temperatures.

### Particle penetration

**Figure 6:**
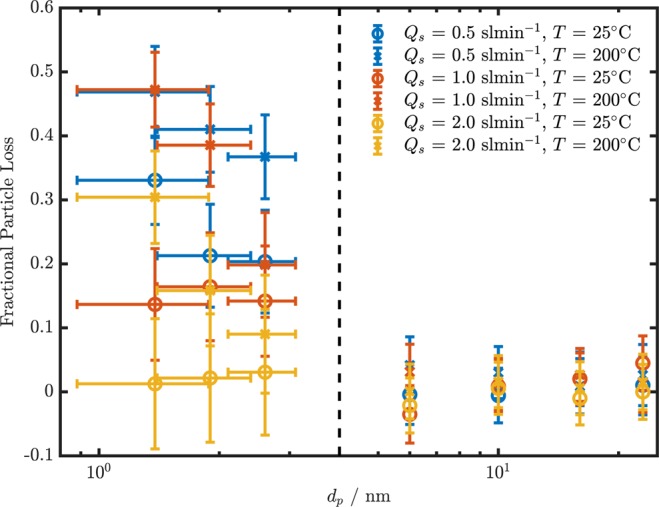
Fractional particle losses as a function of the particle size for different sample flows. The data point to the left of the dashed line (<4 nm) are determined by using the a polydisperse aerosol and the PSM. The data points to the right of the dashed line (>4 nm) are determined using monodisperse aerosol and the DMA-CPC approach.

The particle size dependent fractional particle losses induced by the AGES are shown in Fig. 6. The fractional particle losses are measured and calculated similarly as for the exchange rate of gaseous compounds. The focus is on the sub-23 nm regime, where diffusion is the dominant loss mechanism. The losses of a comparable device in this particle size regime are not covered in the literature to the best of our knowledge. For particle losses at 6 nm, 10 nm, 16 nm and 23 nm were determined for monodisperse aerosol using a DMA and a CPC, as described in the methods section. The particle losses at 1.4 nm, 1.9 nm and 2.6 nm were measured with a PSM for a polydisperse particle-size distribution with a mode at approximately 2 nm.

The particle losses determined with the DMA-CPC approach are below 5% under all tested conditions and particle sizes. No trend for the particle losses at increasing temperatures or flow rates can be identified.

The data measured with the PSM shows particle losses between 1% and 47%. As expected, there is a clear trend for increased particle losses at lower flows and higher temperatures. The particle losses determined at room temperature and a sample flow rate of *Q*_*s*_ = 2 sl min^−1^ are below 5% at all size bins. However, the measurement uncertainties of both particle size and particle losses are too high to draw stressable conclusions except the observations of general trends.

## Discussion

We found that the AGES is a potential tool to be used in several applications where removal of gases is required with simultaneous low losses of nanoparticles. The model describing gas exchange was first verified with oxygen and later studied with several inert gases having molecular masses between 18 u and 135 u. The experimental findings agree very well with the model based on the boundary condition that the mass flux through the SPG membrane is proportional to the difference between the concentrations at the inner and outer walls. However, the model seems to overestimate the removal efficiency of hydrocarbons at elevated temperatures. This unexpected behavior is subject to further investigations. With non-sticky gases, the removal of the lightest compounds can exceed 90% efficiency at the flow rates used. Larger molecules do not have enough time to diffuse away from the sample to reach removal efficiency larger than 50%. Thus, the AGES in the current configuration would work as an efficient drying element (no necessary need for dilution) or it could be used in front of a mass spectrometer to exchange main gases. In several applications, like automotive nonvolatile PN measurement, it is beneficial also to remove larger molecules like e.g. large poly aromatic hydrocarbons (PAH) that tend to be found in the particle phase. AGES could be used to remove gaseous (at 200 °C) large molecules but in the current form the diffusion time is not long enough to reach significant efficiency. For applications requiring high removal efficiency of large molecular masses, a redesign of the AGES would be needed. According to the model derived, better performance could be reached with (1) lower sample flow rate, (2) longer porous glass tube or (3) having several parallel porous glass tubes parts in parallel. The maximal operation temperature of 200 °C is limited by the PTFE sealing between the SPG membrane and the stainless steel housing. The application of more heat resistant materials instead will increase the maximal operation temperature and further extend the range of possible applications of the AGES.

The removal of sticky gases was studied with GSA. The measurements results agreed with the model with boundary condition of zero concentration at the wall. As GSA sticks to walls efficiently, the porous glass part has no additional functionality, rather it is probable and possibly problematic that GSA will be stored on the walls of the porous medium. In applications having high concentrations of GSA, this could lead to periodic regeneration or replacement needs of the porous part.

We identified very small particle losses in the AGES at 6 nm particle size (<5%), and further increased diffusion losses in the PSM size range down to 1.2 nm. Thus, the measured losses are smaller than one would expect based on the results of Hagino^13^, where already 6% losses were detected at 20 nm particle size. In general, one would expect only small losses since the porous glass part is practically a 20 cm long isothermal tube. The AGES particle losses presented here are much smaller than previously presented for thermodenuders^10^ or catalytic strippers^28^. In general, in all of these gas removal systems diffusion is the physical mechanism to collect or remove gaseous compounds but also at the same time collect the smallest nanoparticles. AGES and TD share the trait of the whole surface being active whereas CS systems can have also catalytically inactive sites. The advantage of AGES and TD applications compared to CS is that they are not chemically active, thus the risk of unwanted chemical reactions is lower than in the CS. On the other hand, the advantage of AGES and CS applications compared to the TD is that the removal regime can be sustained isothermal at the elevated temperature whereas in TD cooling is required for the activated charcoal. After the AGES, sample gas can be kept hot or then cooled with a desired method having optimal performance. In our tests, the gas was cooled in an uninsulated metal tube, but also the reference aerosol was sampled the same way. Thus similar thermophoresis was expected and also confirmed by the findings from sampling upstream and downstream locations. In general, if one optimized the gas exchange in the AGES with either having lower sample flow, longer porous glass tube or several parallel sections, one would also see increased losses of nanoparticles. However, an application targeted design of the AGES, can significantly improve measurement results of aerosol experiments by lowering the partial pressure of undesired gaseous compounds without the necessity to dilute the sample.

## Methods

Laboratory experiments were performed to characterize the performance of the AGES. The experiments focused on (1) gas exchange of inert inorganic and organic gases, (2) gas exchange of sticky gaseous sulfuric acid (GSA) and (3) nonvolatile particle loss characterization down to 1.2 nm in particle size.

**Figure 7:**
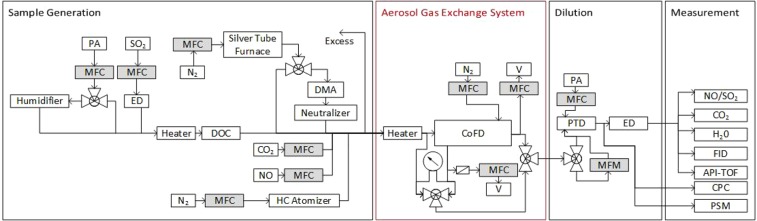
Schematic drawing of the experimental setup.

The measurement setup is presented in Fig. 7. The setup is divided into four parts: sample generation, the Aerosol Gas Exchange System (AGES), dilution and measurement.

### Sample generation

The generation part consisted of a main line, to which silver particles and different gaseous species were injected. By adjusting the main line mass flow controller (MFC) setting, the concentration of species in test was adjusted to a desired value. The main line flow was operated at the range of 3–20 sl min^−1^. The sample humidity/dryness was set by leading the flow either through a humidifier unit or bypassing it.

The gaseous species used for the experiments, their molecular masses and diffusion coefficients in air, typical main line concentrations and the measurement instruments are listed in Table 1. The substances chosen for the experiments were water (H_2_O), nitrogen monoxide (NO), carbon dioxide (CO_2_), sulfur dioxide (SO_2_), ethanol (C_2_H_5_OH), isopropanol (CH_3_CHOHCH_3_), toluene (C_7_H_8_), sulfuric acid (H_2_SO_4_) and *α*-pinene (C_10_H_16_).

The hydrocarbon vapors were generated by setting a flow through an atomizer with a mass flow controller operating at 0.2–0.5 sl min^−1^. The other gaseous species except sulfuric acid were also injected to the main line with mass flow controllers, straight from gas bottles. The gas feeds were changed according to the desired concentration, which depended on the respective measurement instrument limitations.

For sulfuric acid generation a platinum coated diesel oxidation catalyst (DOC)^29^ was used. Upstream the DOC sulfur dioxide was injected to the sample line through an ejector diluter (ED). In the DOC, the sulfur dioxide is oxidized, forming sulfur trioxide, which reacts with water forming sulfuric acid.

$$2{{\rm{SO}}}_{2}+{{\rm{O}}}_{2}\,\mathop{\longrightarrow }\limits^{{\rm{Pt}}}\,{{\rm{2SO}}}_{{\rm{3}}}$$

$${{\rm{SO}}}_{3}+{{\rm{H}}}_{2}{\rm{O}}\to {{\rm{H}}}_{2}{{\rm{SO}}}_{4}$$

Since the aim was to determine the removal efficiency of gaseous sulfuric acid, the sulfuric acid concentration was kept low enough to keep it from nucleating. The particle number concentration was monitored with a condensation particle counter to make sure that only gas phase was present.

For the particle loss measurements, silver particles were generated^30^. A ceramic vessel containing a small amount of silver was placed inside a quartz glass tube, which again was placed inside a tube furnace (Carbolite Gero CWF Model 23 Litre). A nitrogen flow of 2 l min^−1^ was then set through the tube to the main line. Adjusting the furnace temperature allowed for manipulation of the silver particle size distribution, higher temperatures resulting in higher mean sizes. The furnace was operated between 1000 °C and 1200 °C. The particles were fed to the line either straight from the furnace or through a TSI Model 3085 Differential Mobility Analyzer (DMA) coupled with a neutralizer. The neutralizer was used to generate the equilibrium charge distribution of the particles and to consequently reduce electrophoretic particle losses.

### Sampling and dilution

The sample flow through the AGES was created with a combination of a porous tube diluter (PTD) and an ejector diluter. The ejector diluter was operated with a dilution air pressure of 1–2 bar. Changing the pressure allowed for manipulation of the secondary dilution ratio and the ejector inlet flow. The PTD dilution air flow was then chosen accordingly to reach the desired sample flow through the AGES. The sample flow was measured with a TSI model 4140 mass flow meter. The flows chosen in the measurements were 0.5, 1.0 and 2.0 sl min^−1^. The valve system of the measurement setup was built so that it was possible to draw sample before the AGES, after it and from the purge gas flow.

### Measurement instruments

The instruments used for gaseous species concentration measurements were LI-COR LI-840A for CO_2_ and H_2_O, SIDOR gas analyzer for SO_2_ and NO and an Atmospheric Pressure Interface Time Of Flight (API-TOF) mass spectrometer^31^ for gaseous sulfuric acid. The API-TOF was operated with an Eisele-type nitrate inlet for chemical ionization^32^. Baseline Series 9000 NHMC flame ionization detector (FID) was used for the measurement of hydrocarbons. The FID has a different response for each different substance, but as the quantity to be determined was the removal efficiency (and not absolute concentration) for each substance, the response difference does not interfere with the data analysis.

For total particle number concentration measurement an Airmodus A10 Particle Size Magnifier (PSM) coupled with an A20 CPC and a TSI CPC model 3775 were used. The PSM allows for manipulation of the saturator flow and thus the cutoff size of the PSM-CPC pair. Periodically changing the saturator flow allows for calculation of particle concentrations in the size bins determined by the chosen saturator flows. In this measurement, saturator flows of 1.22, 0.298, 0.191 and 0.102 sl min^−1^ were used, which correspond to cutoff sizes of 1.17, 1.63, 2.21 and 3.07 nm, respectively.

### Test matrix

The substances presented in Table 1 were chosen to get a representation of both inorganic and organic molecules and to have variation in molecular mass and vapor pressure to see if it has an effect on the species’ storage on the porous glass surfaces.

### Measurement procedure

Concentrations of the gas species were measured upstream and downstream of the AGES for the removal efficiency determination. The measurement procedure also included a dilution air background measurement, which meant sampling only dilution air. This was done to monitor drifts in measurement instrument zero levels and to correct the concentrations accordingly in the data processing phase. All measurements were conducted with a hot system (AGES heaters at 200 °C) and a cold system (AGES heaters off), with the exception of GSA, with which only the hot system was used. For the most part, the measurements were made for one substance at a time, but H_2_O, CO_2_ and SO_2_ were also coupled with other substances.

The GSA concentration had to be kept low in order to prevent it from nucleating. The appropriate concentration was found by adjusting the SO_2_ feed, main line flow and flow through the humidifier until no particles were detected with the CPC. It was observed that in order to prevent the nucleation a very low concentration of GSA was required. Because of this the background signal was approximately half of the measured signal. The background signals for the sampling points were measured by sampling only air from the main line and it was observed that the background signal was different for each sampling point (upstream and downstream). The reason for this can be that even with the DOC heaters and SO_2_ feed turned off there was some trace amount of GSA released from the main- and sampling lines or the DOC. For this reason, background signal levels of all sampling points were measured to calculate the removal efficiency accurately.

Due to relatively long integration times (>5 min) and accurately mass flow controlled sources of substances the measurement uncertainty for gas measurements is found to be dominated by the sample flow measurement. The uncertainty of this measurement amounts to 0.05 sl min^−1^.

### Particle loss measurements

We used two different experimental approaches for the determination of particle size dependent losses. The first approach was to inject monodisperse particles and calculate the losses from concentrations measured by a TSI CPC 3776. Particles of 6, 10, 16 and 23 nm in diameter were classified using a TSI model 3085A Nano DMA and injected into the main line. Observations of the particle source showed that the particle number concentration varies by approximately 2% between two consecutive measurements that were used to determine the particle losses. The uncertainties for the fractional particle losses were calculated from the uncertainty caused by the particle source variation, the Poisson counting statistics uncertainties and the application of Gaussian propagation of uncertainties.

The second approach was to inject polydisperse particles from a tube furnace and calculate the losses from size distributions measured by the PSM upstream and downstream the AGES. This transient measurement method enables the measurement of smaller particles but exhibits higher measurement uncertainties than the approach with monodisperse aerosol.

The uncertainty of the PSM particle size bins positions is subject to a number of effects including particle composition dependent detection probability, CPC calibration with differently charged particles and limited DMA resolution due to Brownian motion^33^. The combination of these effects is estimated to yield an uncertainty of ±0.5 nm.

We observed that the generated particle number concentrations vary by approximately 5% between two successive measurement points. This uncertainty, which is caused by the particle source is added to the Poisson uncertainty for the determination of the uncertainty of the fractional particle losses.

## Data Availability

The data sets and codes used and analysed in this study are available from the corresponding author on reasonable request.
